# Factors influencing the clinical decision-making of midwives: a qualitative study

**DOI:** 10.1186/s12884-017-1511-5

**Published:** 2017-10-06

**Authors:** Darie O. A. Daemers, Evelien B. M. van Limbeek, Hennie A. A. Wijnen, Marianne J. Nieuwenhuijze, Raymond G. de Vries

**Affiliations:** 1 Research Centre for Midwifery Science Maastricht, Zuyd University, PO Box 1256, 6201 BG Maastricht, The Netherlands; 2 Caphri School for Public Health and Primary Care, Maastricht University, PO Box 1256, 6201 BG Maastricht, The Netherlands

**Keywords:** Midwife, Midwife-led, Evidence based medicine, Clinical decision-making, Qualitative research, Pregnancy, Childbirth, Maternity care, Vignette, Woman-centred

## Abstract

**Background:**

Although midwives make clinical decisions that have an impact on the health and well-being of mothers and babies, little is known about *how* they make those decisions. Wide variation in intrapartum decisions to refer women to obstetrician-led care suggests that midwives’ decisions are based on more than the evidence based medicine (EBM) model – i.e. clinical evidence, midwife’s expertise, and woman’s values - alone. With this study we aimed to explore the factors that influence clinical decision-making of midwives who work independently.

**Methods:**

We used a qualitative approach, conducting in-depth interviews with a purposive sample of 11 Dutch primary care midwives. Data collection took place between May and September 2015. The interviews were semi-structured, using written vignettes to solicit midwives’ clinical decision-making processes (Think Aloud method). We performed thematic analysis on the transcripts.

**Results:**

We identified five themes that influenced clinical decision-making: the pregnant woman as a whole person, sources of knowledge, the midwife as a whole person, the collaboration between maternity care professionals, and the organisation of care. Regarding the midwife, her decisions were shaped not only by her experience, intuition, and personal circumstances, but also by her attitudes about physiology, woman-centredness, shared decision-making, and collaboration with other professionals. The nature of the local collaboration between maternity care professionals and locally-developed protocols dominated midwives’ clinical decision-making. When midwives and obstetricians had different philosophies of care and different practice styles, their collaborative efforts were challenged.

**Conclusion:**

Midwives’ clinical decision-making is a more varied and complex process than the EBM framework suggests. If midwives are to succeed in their role as promoters and protectors of physiological pregnancy and birth, they need to understand how clinical decisions in a multidisciplinary context are actually made.

## Background

A defining feature of midwifery care is the promotion and protection of physiological reproductive processes [1]. During the course of pregnancy and childbirth, midwives are constantly weighing the appropriate care for each individual woman, including when the assistance of specialised caregivers is needed. This assessment demands well-developed competencies for clinical decision-making [2]. Ideally, midwives provide evidence-based care, using the best available clinical evidence, their own clinical expertise, and the situation and values of the pregnant women [1, 3]. However, we know that midwives’ intrapartum referral decisions differ and that this cannot be explained by medical circumstances or women’s characteristics alone [4–6]. This suggests that other factors may be involved in the clinical decision-making process.

Cheyne et al. [5] identified three elements in the decision-making process of midwives and obstetricians: the *assessment* (the professional’s judgement of the level of risk), the *decision* (the choice between possible courses of action) and the *decision threshold* (the professional’s threshold when linking the judgment and the decision). They found that although midwives and obstetricians made similar case *assessments*, there was great inconsistency with regard to referral *decisions* within the groups. This suggests that the main source of variation is in the personal *decision thresholds* of professionals [5]. Several studies suggest that factors related to the individual midwife contribute to the variation in decisions, such as: experience of earlier adverse events, definition of the boundaries of physiological birth, perceptions of risk, methods of managing the uncertainty during the childbirth process, practice philosophy, attitude towards collaboration with other professionals, and interaction with the woman [4, 7–11]. Intrapartum referral rates are also affected by features of the midwife-led practices and the local infrastructure such as number of midwives working in the practice and the distance to the hospital [5, 9, 12]. Studies among other health professionals confirm the diversity of factors affecting a clinical decision [13, 14]. Clinical decision-making thus seems to be a more complicated and less rational process than suggested by the definition of evidence based medicine (EBM).

Most research on decision-making in midwife-led care is done quantitatively with a focus on the intrapartum decision and not on *how* the decisions were made. Our research addresses this lacuna, offering insight into factors that influence midwives’ clinical decision-making in pregnancy and childbirth by means of in-depth interviews with practicing midwives.

## Methods

### Design

We undertook a qualitative study using in-depth interviews. Each interview started with the exploration of authentic written cases, vignettes, followed by a semi-structured interview. The Vignette Method is especially suited to explore people’s judgements, perceptions, attitudes, potentially sensitive topics, accounts of practice, and influencing factors [15, 16]. Midwives were invited to come to a clinical decision in each vignette and to verbalise their thoughts. This approach, the “Think Aloud” method, allows description of the points of information that are concentrated on and how information is structured during a problem-solving task [17] and provides rich and extensive data for analysis [18]. We constructed three vignettes including situations in pregnancy, childbirth, and puerperium. Two vignettes consisted of two or more phases (Table 1).

**Table 1:** Vignettes

	Phase	Content
1	1	Pregnancy
		Gravida 2, para 1, aged 35, BMI 36, 28 weeks gestation, caesarean section in history. Obstetrician decides on a referral to obstetrician-led care after a routine consult at 30 weeks because of caesarean section in history. Obstetrician’s ground: obesity
	2	Puerperium
		2 days postpartum: the woman asks the midwife to inject a thrombo-prophylacticum prescribed by the hospital.
2	1	Pregnancy
		Gravida 2, para 1, aged 32, BMI 32, 20 weeks gestation, under care of a neighbouring midwifery practice where homebirth is no option although this is the woman’s preference. The woman is discontented and asks for a switchover to your practice and your opinion of a homebirth.
3		Birth
		Gravida I, para 0, aged 26, 40 weeks 3 days’ gestation.
	1	1) Partner is calling: his wife has contractions
	2	2) First visit of the midwife
	3	3) 10 h after the first contact (partogram)

Obesity was introduced in the vignettes because this characteristic challenges midwives’ clinical decision-making on medical and psychosocial levels. Obese women experience stigma, which may threaten the bond of trust between midwife and woman [19] and in the Netherlands, there is scant evidence on the best care for obese women in midwife-led care, clear national guidelines are lacking, and local protocols are ambiguous [20]. Based on discussions with practising midwives (other than the ones interviewed), we identified typical clinical dilemmas associated with supporting obese women and incorporated these in the vignettes.

**Fig. 1:**
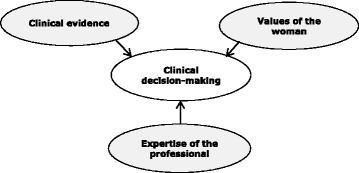
Evidence based medicine

Because clinical decision-making is, in part, an unconscious process [21] we continued the appraisal of vignettes using a semi-structured interview in order to make all influencing factors as explicit as possible. We choose for individual interviews to avoid peer influence on a midwife’s answers and to allow fundamental exploration of an individual midwife’s perceptions and motives. For the interview, a semi-structured interview guide was developed (Table 2), based on the theoretical framework of EBM (Fig. 1).

**Table 2:** Interview guide semi-structured interview

Interview guide
1. You have to make a clinical decision in your care for a specific woman: What do you take into account? Which aspects do you consider?
2. What sources of knowledge do you draw on in making your clinical decisions?
3. What is the role of a woman’s characteristics in your clinical decision-making?
4. Do you explore women’s preferences and how do you manage them in your clinical decision-making?
5. How does your clinical expertise influence your clinical decision-making?
6. Are there specific features of your personality that may influence your clinical decisions?
7. What is your attitude towards midwifery and how does that attitude influences your clinical decision-making?
8. Are there aspects of your clinical decision-making that we have not discussed and that are important to add?

The internationally recognised model of EBM is defined as the conscientious, explicit, and judicious use of best available evidence in making decisions about the care of individual patients. In its ideal form, the practice of evidence based medicine integrates the clinical expertise of the professional with the best available external clinical evidence from systematic research while taking into account an individual patient’s situation, predicaments, rights, preferences and values (Fig. 1) [3].

The research team included five members: two professors in midwifery science, two PhD’s (one health promotion and one midwifery science) and one PhD student and midwife (PhD at present). The interviews were done by the first author, who has a long history in midwifery education and guideline development.

### Setting and participants

A total of 11 interviews were conducted between May and September 2015 with midwives working in primary care, midwifery practices across the Netherlands. Since midwife and practice characteristics may influence the clinical decision process [4, 5, 7–10, 12], we choose purposive sampling to achieve representative variation with gender, age, years of midwifery experience, highest level of education, practice characteristics, and features of the practice population as main criteria. Midwives were invited to participate by e-mail followed by a phone call. We began with six midwives and continued recruitment until saturation was reached [22, 23]. After nine interviews we reached saturation on the level of themes and subthemes, but we did two additional interviews for confirmation. Only one midwife declined to participate because of workload.

### Data collection

Before the interview, participants filled out a short questionnaire on their demographic characteristics. The interview was conducted by the first author at the participant’s preferred location (home or in the midwifery practice). It started with a short introduction of the vignettes and the Think Aloud Method [17, 18]. The vignettes were presented to the midwives one by one and phase by phase and interaction was limited to clarifying questions and encouragement to think aloud. Subsequently, the interview continued based on the semi-structured interview guide, and midwives were encouraged to introduce any issue related to the topic of the study. The interview was concluded with a short evaluation and field notes describing the context of the interview and the participant. The interviews lasted between 1 h 20 min and two hours.

### Data analysis

All interviews were audiotaped and transcribed verbatim by the first author and an assistant. A thematic analysis was performed using QSR NVIVO 8 [24, 25]. A preliminary coding scheme was developed by the first (DD) and second author (EvL) based on the framework of the interview guide and the data of three, randomly chosen, interviews, that were, coded by the first and second author independently. The final coding scheme emerged during further analysis based on consensus. Transcripts were coded by the first author who presented her analysis to the research team. Codes were grouped into subthemes and themes by examining the commonalities, differences and relationships within and among the interviews and through reflective discussion with the research team [26].

We used the following strategies to ensure the rigour of our study: Vignettes were made in accordance with recommendations for vignette construction [15, 16, 27] and were reviewed by four midwives working in the field of education, research, and midwifery practice. After every interview we asked the participants to comment on the content and authenticity of the vignettes. The first interview was organised as a pilot followed by an extensive evaluation of the process and the content of the interview with the interviewee and an observing researcher (HW). The combination of the Vignette method, Think Aloud procedure, and semi-structured interview aimed to obtain a complete range of data on the topic (methodological triangulation). Throughout the study several researchers reflected on the analytic process (investigator triangulation). Research team meetings were organised regularly to discuss the scientific and organisational aspects of the study (peer debriefing). The translation of the quotes was assisted by a native English speaker. The whole procedure of the study was recorded in a logbook. The writing of this article was guided by the consolidated criteria for reporting qualitative research (COREQ) [28].

## Results

Table 3 shows characteristics of the participants. Ten midwives worked in midwife-led practices. One midwife worked in an integrated care system where a midwife-led care unit exists alongside the obstetrician-led care unit and where all midwives work in both settings during the same shift.

**Table 3:** Characteristics of the participants

Characteristics	*N* = 11
Gender	
male	1
female	10
Age (mean and range)	43.8 (28–54)
Year of graduation	1983–2012
Years of experience (mean and range)	19.6 (2–32)
Highest level of midwifery education	
Bachelor in midwifery 3 years	5
Bachelor in midwifery 4 years	4
Bachelor of science in midwifery	1
Master of science in midwifery	1
Midwifery-related responsibilities outside of the practice	
Yes	7
No	4
Characteristics of the practice^a^	
Number of registered women yearly	40–525
Number of midwives	1–6
Number of registered women per midwife	98 (40–158)
Duration of being on call	24–56 h, 1 caseload midwife
Mean working hours per week^b^	30–60
Practice population mainly typified as	
-regarding level of education-:	
Low	1
Middle	6
High	2
Mixed	2
-regarding ethnicity-:	
Dutch	6
Dutch and Western immigrants	3
Dutch, Western and non-Western immigrants	2

^a^1 additional midwife works in an integrated care system in a hospital together with 9 colleagues in 8 h shifts; about 550 women are registered yearly

^b^also including not-woman related tasks

**Fig. 2:**
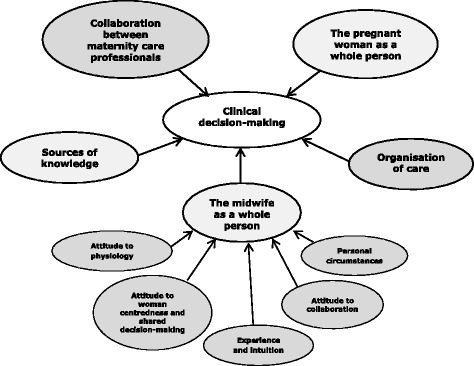
Clinical decision-making process: emerging themes and subthemes

We identified five themes that influenced everyday clinical decision-making: the pregnant woman as a whole person, the midwife as a whole person, sources of knowledge, collaboration between maternity care professionals, and organisation of care. Looking more closely at the midwife, we found five characteristics that shaped her decisions (Fig. 2).

### The pregnant woman as a whole person

During the interviews it became clear that midwives used a whole person approach in their work with women. All gathered relevant physical information of the pregnant woman described in the vignettes through history taking, observation, conversation, exploration of woman’ symptoms and diagnostic tests.

*Of course, I’m going to sit quietly together with them [woman and partner], observe the nature of the contractions, let them hear the heartbeat, external examination, vaginal examination: what is the dilatation (…) (Midwife10)*

But midwives also paid attention to the woman’s psycho-social context: her thoughts and feelings, the relationship with the partner, the socio-economic situation, the supportiveness of the woman’s social network, her lifestyle, and the media influencing women’s opinions.

Psycho-social context:

*Woman’s characteristics, I think that everything about the woman is important for decision-making, the total picture counts (Midwife6)*

*Disadvantaged social environment, low intellectual abilities, hypochondriac, women with anxiety disorders, you take that all into account. Women who had a very traumatic experience the first time … (Midwife1)*

Relationship to the partner:

*If partners sometimes have no connection with each other and are getting in each other’s way, that makes me sometimes think: I need some help here [=reason for moving from home to hospital setting] (…) (Midwife9)*

The media:

*A lot of people were highly influenced by journalism (…) when there was almost weekly in the papers that, um, homebirth was irresponsible, that [promoting physiology] was a very difficult message (Midwife5)*

Midwives reported that they took into account the preferences of the women and that they strived for satisfied women with good childbirth experiences. As result midwives made efforts to balance their basic attitude towards guarding physiological birth and the wishes of women for medical interventions, such as the use of epidural anaesthesia for pain relief.

*Yes, I want to give everyone the opportunity for a natural, healthy, good childbirth: that is my aim. But (…) if you need a medical intervention to, um, have a satisfying and good experience, and of course to have a healthy child, yes that also fits in and in that process I am the one who has to support, who has to perform an appropriate risk assessment. Yes I hope that the woman connects with me during pregnancy and childbirth; that she trusts me and, um, that we together make the best choices and that the birth goes well and is safe (Midwife11)*

### Sources of knowledge

Midwives indicated that they initially gathered their professional knowledge, the ground for clinical decision-making, during their midwifery education. They also pointed to their preceptors as important role models.

*(I gathered my professional competencies) during my education (…) I think that the examples I had were the midwives who very much went into conversation with the women (Midwife3)*

Midwives updated their basic competencies in different ways: by reading the national midwifery journal, by attending continuing education courses, by using the internet, by consulting colleagues, by participating in working groups. (Inter)national guidelines were reported as sources of knowledge but almost all midwives emphasised their use of the local protocols. Regarding obesity, these protocols vary in their determination of when a midwife-led hospital birth is appropriate.

*That is a strict policy here (…) with a BMI of 32, a midwife-led hospital birth is advised (…) (Midwife8)*

*A midwife-led hospital birth [is advised] for a BMI of 35 or greater (…) a BMI of 32 is for us no reason to advise a birth at the hospital (Midwife7)*

Midwives varied in their adherence to the guidelines: from strict use to a critical appraisal of the applicability of the guideline together with the woman. This variation was also visible in their role regarding the construction of the local protocols: from an implicit faith in the quality of the protocols to an active and critical contribution to the content of the protocols.

*We are rather strict, um, in the adherence to the guidelines, um, yes we find that important (…) we agreed with the recommendation, um, certainly when there are risks (…) (Midwife8)*

*I really see them as guidelines (…) that you can deviate in individual cases in agreement with the obstetricians, on the condition that you communicate well and report everything, I make the decisions but I confer on everything [with the obstetrician] (Midwife3)*

*No, I trust that the protocols (…) really are well-founded (…) and if there is a concept, then I read it and I think: yes, that looks good, I can work with this, I agree with this but the evidence behind it? No, I’m honest: I never did ask for or look at it (Midwife1)*

In addition, some midwives mentioned local perinatal audits – meetings where maternity caregivers critically analyse the care given in cases of perinatal morbidity or mortality [29] – as an important source of knowledge.

*I have to say that the audits (…) very much help me (…) that you think differently, work differently, [I learned] that you must be able to justify all the decisions you make (Midwife11)*

### The midwife as a whole person

Several characteristics of the midwife – both personal and professional – play a role in her decision- making, including: her attitude towards physiology, woman centredness and shared decision-making; her experience and intuition; her attitude toward collaboration; and her personal circumstances (Fig. 2).

#### Attitude to physiology

All interviewees said it was important to guard the physiology of pregnancy and childbirth. The midwives informed women that pregnancy and childbirth are natural and normal processes. They also described a number of interventions to support physiological pregnancy and childbirth, focussing on the empowerment of the woman in order to reinforce her capacity to give birth. These interventions included supporting the woman with a quiet environment, breathing techniques, varying positions and different tools in labour and childbirth. Midwives also explained that efforts to protect a physiological approach to birth may require profound discussions with obstetricians, as in the case of differing opinions on the necessity of a transfer of a woman to obstetrician-led care

*I don’t do much, I, um, especially try to help to relax, um, that there is no stress, that women surrender themselves to the birthing process. I try to avoid stimuli in the woman’s environment: I darken the room, make it quiet, I try to talk quietly. I try to help her with breathing quietly, with avoiding resistance to the process, with finding relaxation between the contractions. I suggest to take a bath, to shower, yes, you don’t do very much (…) if she has a lot of pain, yes, we give sterile water injections (…) we have good experiences with this, (Midwife11)*

*That women have to trust their body (…) and that I can give them that trust through my information, my attitude, my things, that I can help them the best with that (Midwife7)*

Responding to one of the vignettes that described a transfer to obstetrician-led care at 28 weeks (Table 1, vignette 1, phase 1), one midwife said:

*The local agreement is that she [pregnant woman] is referred to the care of an obstetrician at 36 weeks of pregnancy because of a caesarean section in history, thus [an obstetrician- initiated] takeover of care at 28 weeks is not needed (…) thus I certainly would bring this up with the obstetrician, I would make a phone call (Midwife3)*

On the other hand, the interviews also revealed fundamental differences between midwives in the extent of their support for protecting the physiology of pregnancy and childbirth. We found differences in: the use of interventions which are proven to support physiological birth such as continuous support in labour; attitudes towards applying diagnostic tests or interventions in general such as ultrasonography or rupture of the membranes; perceptions of certain situations or women’s characteristics as risky and in the handling of those perceived risks; appraisals and application of guidelines; support for homebirth. This results in a variation from midwives who are always looking for the most physiological approach possible – irrespective of guidelines or organisational hindrances – to midwives who use extra diagnostic tests or are quick to consult secondary care in order to reassure themselves or the women and their partners.

*Yes, we give continuous support - if a person prefers that- in the active phase (Midwife11)*

*What we do with continuous support? Honestly, we are not so used to that in our practice I must confess and, um, if women indicate: gosh this is too much for me (…) then I stay with them [in their home] but it is not a standard procedure (…)(Midwife9)*

*In case of a BMI ≥ 35 we advise a midwife-led hospital birth; when women want to have a homebirth we explain that there are some risks such as more blood loss (…) but if women really want a homebirth despite the information (…) if someone gave birth the first time without any blood loss problem, I do not expect a postpartum haemorrhage just because of a BMI of 37 (…) it is no problem for me to support this homebirth (Midwife5)*

*Of course I try to reassure them but if persons stay worried then I offer: ‘if you prefer then I arrange something, then you can undergo a CTG in the hospital, they check your blood pressure or whatever – depending what is going on - (…) then you are totally reassured’. Yes, I take care that women and their partners leave the practice satisfied and if that means a consultation with the obstetrician: fine (Midwife4)*

Midwives experienced dilemmas in their pursuit of physiological birth. They struggle with the changing cultural ideas about birth over time.

*[Earlier] I sometimes worked with a woman during 3 or 4 days, with a prolonged labour, because I was afraid that if I should refer her, I was told that I did not coach her enough (…). Now it is the opposite situation, I got a complaint because she was unsatisfied because she did not get pain reduction. ‘Did you have a bad experience?’ ‘No, I just wanted to have that pain reduction’ (Midwife11)*

In this context, the most important dilemma that midwives experienced was how to deal with the difference in opinion about the nature of physiological obstetrics between midwives and obstetricians. Midwives felt pressured to refer to secondary care earlier. This was demonstrated by the differing opinions on guidelines between primary and secondary care and by discussions about how to make room for a more physiologic approach.

*We think (…) that the process of the development of care pathways is guided too much by secondary and even tertiary care [professionals], yes, um, we think that physiology has been lost sight of (…) . We [midwives in that working group] have tried very hard to have an approach based on the practice of homebirth and physiology. Um, yes, on little components we succeeded somehow but that has been a really tough process (…) that means that a mother’s third child of 3000 grams - born at home - should be admitted to the hospital for a 24-hours- glucose-protocol. That’s going much too far for me and I will not go along with that (Midwife7)*

*Yes, I think that the pressure by the secondary care plays a big part in clinical reasoning and decision-making. Um, the pressure you feel from the rules, from the disciplinary tribunals that have been, that has resulted in more constraint within our profession, I think. Due to this you are even more inclined to call the obstetrician for certainty, do a consultation for safety’s sake (…) but I do not want to go along with this (Midwife6)*

#### Attitude to woman centredness and shared decision-making

While all midwives in our study sought to consider the preferences and needs of women in their decision-making, they varied in the extent to which they put woman-centredness into practice. For example, midwives differed in the way they involved the woman in making decisions. This ranged from those who discussed everything with the woman and offered her real and acceptable choices to midwives who used the guidelines to direct decision-making. In between these extremes were midwives who relied on their intuition and their knowledge of the woman in assessing how to support her or what to do.

*That is what I tell to women: concerning your BMI, this is the guideline: what do you think of it? What do you want? If it concerns a BMI≥40, I say: following the guideline, I have to refer you to the obstetrician. There are two different women: one says OK. The other says: I don’t need this, I’m healthy, I feel healthy, I’m pregnant, I don’t want to go to the obstetrician. In that case, we are going to talk (…) I’m going to explain the risks related to BMI (Midwife6)*

*I think I do it more intuitively, yes and (…) I know my women, I saw them throughout pregnancy. Then you do it intuitively: what kind of woman is this? Um, I look at the situation when they are in labour; um, I also sense a bit of what is possible (…) (Midwife2)*

*No, we try to adhere to the guidelines, um, you are part of [local collaboration between midwives] and you all want to handle patients equally. It cannot be possible that the patient can go ‘shopping’ [between midwifery practices] and thinks: ‘in that practice I can have a homebirth’. I wonder: if you are stretching your boundaries, where is the end? (Midwife8)*

Midwives experienced another dilemma in this context: as long as the preferences of a pregnant woman could be achieved within the boundaries of primary care as locally agreed upon, midwives were willing to meet them. If a woman’s preferences exceeded the responsibility and scope of primary care midwives, they were less willing to oblige. Because local protocols are created in dialogue between local primary and secondary care professionals, they often differ between localities, and therefore midwives’ dilemmas regarding this issue also differ by location.

#### Experience and intuition

All midwives agreed about the important - but sometimes unconscious - role of experience in clinical decision-making. Their experience provided know-how and routine (pattern recognition) and made them feel certain. Midwives with a long career have seen a lot of trends and opinions, have learned from it and remain nuanced in the face of a new ‘hype’. Experience also has another dimension: midwives sometimes keep negative experiences in mind, resulting in more defensive management when similar situations arise. A midwife reported that she was influenced by her own childbirth experience in making clinical decisions.

*We had a lot of referrals for pain relief (…) at that time we were very busy with the concept of active support of labour: all women had an amniotomy at a dilatation of 3-4 cm. I thought: Yes, of course, those women are in the beginning of labour, it is tense for them, they have contractions every 3 or 4 minutes. You rupture the membranes and they experience acceleration, women are scared to death, no wonder that they ask for pain relief. Thus we don’t do that anymore (…) I think that I very often allow my experience to play a part especially in the ‘grey area’ [boundary between physiology and pathology]. You know: maybe I am somewhat less cautious, um, that indeed I more easily can deviate from the guidelines because of my experience (Midwife2)*

*That is particularly with postpartum haemorrhage (pph): if you just experienced 2 pph, with woman number three you are more likely to give extra oxytocin…. So yes…clinical management based on the past (Midwife10)*

All midwives stated that intuition plays a part in clinical decision-making, especially in combination with a gut feeling that something is wrong. In exploring the phenomenon of intuition with the interviewees we learned of situations where small deviations in the physiological process combined with their professional expertise and their in-depth knowledge of the pregnant woman enabled them to perceive ‘other’ behaviour or subtle symptoms. Although the midwife could not (yet) transform the deviations into a diagnosis, she took action.

*Midwife (M): was this woman going to have a pph? My feeling said: give her 5E Oxytocin extra (…) but I had nothing to go on, that was purely my feeling (…)*

*I: (…) but what do you take into account?*

*M: (…) the course of the delivery, the amount of blood loss until now, the time between the birth of the placenta and the blood loss, the quality of the contractions of the uterus, is there perineal damage, do I expect a rupture of the cervix, did I have incidentally another two pph today, that I surely consider and um perhaps also a bit: it doesn’t hurt to try in this case (Midwife10)*

#### Attitude towards collaboration

All midwives endorsed the importance of a good collaboration with obstetricians but the personality of the midwife determined how they approached that collaboration. This was illustrated by the difference in midwives’ communication during interprofessional consultation. Ranging from handing over control to a constructively critical dialogue with obstetricians when needed. On one hand, some midwives seek dialogue-based consensus, and on the other hand, some aim to please women and colleagues and avoid discussion. In this example, a midwife cedes control to the obstetrician by asking for permission to keep the woman under her care:

*Then I would consult the obstetrician (…):* may *I wait and see or do you prefer the referral now? (Midwife1) [emphasis added]*

While this midwife challenges the advice of the obstetrician:

*For example, the guideline on diabetes: all the different reference values [in the different (inter)national guidelines], makes it clear to me that (…) if the obstetrician says very definitely: this woman has diabetes and needs to be referred, I say: but this is not so definite, there is room here and this woman has a special wish and she wanted to do this in her way, can we collaborate on this? (…) I try it every time (Midwife6)*

#### Personal circumstances

Finally, midwives described how personal characteristics, feelings and conditions can influence clinical decision-making.

*If I have had a tough shift then my perception at the end of this shift is different, I take that into account, (…) yes, you are influenced by your own state of mind and the things you find very thrilling, these also influence you (Midwife9)*

### Collaboration between maternity care professionals

Midwives working within one practice aim for continuity of care for pregnant women. However, individual midwives may differ from their colleagues in their clinical decision-making in particular situations. Midwives’ response to within-practice variation is to make general agreements and/or to discuss individual cases.

*We have a colleague who sometimes finds it [homebirth] hard and the agreement is clear that we do not decide upon a homebirth in advance. The midwife who is responsible during childbirth decides if she feels up to the task (Midwife5)*

Midwives and obstetricians cooperate locally and the nature and quality of this cooperation influence midwives’ clinical management. Midwives described this local collaboration as positive when it was respectful and based on equality; when their view of physiological pregnancy and childbirth was assessed at its true value by obstetricians; when primary and secondary care professionals did not have strongly divergent perceptions of risk; when well-reasoned deviation from the guidelines was accepted; and when dialogue was possible in situations where pregnant women challenged the primary care boundaries. Personal relationships between professionals or groups of professionals also determined the nature and the quality of the collaboration. Importantly, when midwives worked with two different hospitals they were able to use the professional and personal differences between the institutions to their advantage.

*I’m thinking in terms of cooperation, to have respect for each other’s responsibility and way of working. We have to strive for more joint policy making and I accept that with open arms (…) it is fine to me that an obstetrician calls me and asks ‘why you did this?’, I find this pleasant, we can learn something from that (…) but not pointing the finger or um that is not a pleasant collaboration to my opinion (Midwife11)*

*It is convenient to have different hospitals [to work with, with different protocols] which enable you to advise women: I would do that in hospital X and that in hospital Y (Midwife5)*

In this theme, the other themes we observed converged: in positive collaborations there seemed to be more room for physiological aspects of birth and woman’s preferences. In case of difficult collaborations, medical thinking and local protocols dominated and midwives had to have a more assertive personality and good scientific knowledge in order to realise women’s preferences and physiological birth.

### Organisation of care

The interviews revealed how the organisational aspects of care influenced clinical decision-making. For example, legislation regarding the working conditions for ambulance drivers forbids lifting persons above a certain weight. This limits homebirth and was explicitly taken into account in midwives’ clinical decision-making.

*In our area it is above 100 kg (…) if they live in an apartment building [with no elevator] than they have to give birth on the ground floor (…) that is the local agreement because of the lifting of women [by the ambulance drivers] (Midwife9)*

Considerations of competition between midwifery practices also play a part in decision-making. Where midwifery practices offer non-medically indicated ultrasounds to women, other practices in the area feel pressured to do the same. Also the quality of medical facilities, financial considerations, the hour of the day, and busy shifts in a midwifery practice or in the referral hospital influenced decision-making.

*Sometimes you enclose the hour of the day in your decision (…) or the weekend (…) or if you live somewhere um a quarter of an hour driving um you include that in your decision (Midwife4)*

## Discussion

This study gives us a more complete view of the factors driving the clinical decisions of midwives. Theoretically, evidence based decision-making rests on three pillars: clinical evidence, the expertise of the professional, and the values of the woman. In our study we found these pillars did play a role in the themes ‘sources of knowledge’, ‘the midwife as a whole person’ and ‘the pregnant woman as whole person’. With regard to the midwife, however, clinical decisions were influenced by far more than her expertise (e.g. her education, experience, and intuition). Her attitude towards physiology of birth, woman centredness, shared decision-making, and collaboration, as well as her personal circumstances helped to shape her decisions. Two additional factors – ‘collaboration between maternity care professionals’ and ‘organisation of care’ – also played a role. Our findings correspond with those from other studies, confirming that clinical decision-making is a more varied and complex process than the EBM framework suggests [4–14, 30].

We also found that the clinical decision-making of midwives was influenced by the nature and content of the local collaboration with maternity care professionals. Although midwives and obstetricians share the goal of providing the best care for mother and child, their collaborative efforts to achieve this goal are challenged by their different philosophies of care and different practice styles [31–33]. Downe et al. [34] suggest that the trend toward risk aversion and the medicalisation of childbirth may exacerbate this polarisation between obstetricians (who typically support this trend and midwives (who typically resist it). We observed that midwives struggled with this inter-professional tension in their collaborations with obstetricians. Like O’Connell et al. [35] we found their reactions to the tension varied from acquiescing to the system, to living with the conflict, to rebelling against the norms of practice.

In cases of referral or consultation with an obstetrician, midwives felt the need to account for their interventions, and even more, for their decisions to *withhold* an intervention, a phenomenon observed by others among midwives working in hospital settings and among community midwives, whose independent clinical decision-making is often challenged [36–38]. Since a well-defined philosophy of care and a supportive environment are described as major factors contributing to effective and respectful clinical decision-making, it is the responsibility of both midwives and obstetricians to create the kind of collaborative relationships that will safeguard the rights of women [39].

In 2009, the Dutch government published a report ‘On safe care of pregnancy and childbirth’ [40] in response to a perceived problem of high perinatal mortality rates in the Netherlands [41]. An important recommendation was to reinforce local collaboration among primary, secondary and tertiary maternity caregivers. A few years later, the Ministry of Health proposed a reorganisation of the ‘stratified’ – i.e., primary and secondary – model of care into an ‘integrated’ system. This development – which presented both new opportunities and new threats for midwifery care – forms an important backdrop for our study and helps to explain our findings.

Local protocols – which were a key factor in shaping our respondent’s clinical decision-making – are a product of this newly intensified local collaboration. As we noted in our study, the recommendations regarding care for obese women are different in different localities, since in the absence of evidence, protocols are established based on consensus between professionals. At this point, differences in risk perception and in philosophy regarding physiological childbirth *between* obstetricians and midwives but also *among* midwives and obstetricians play an important role in prescribed pathways for care.

Midwives are involved in both the creation and the implementation of these protocols. Some, but not all, of participants were actively involved in the - interdisciplinary - writing of protocols. Those who were involved were not always pleased with the quality of the process or the results. We discovered that the nature of the collaboration played an important role in shaping the local protocols: when there was an equal and constructive collaboration, a positive attitude toward promoting physiology, and little inter-professional difference in the perception of risk, there was more room for a physiological approach to care. In addition, the midwife’s professional knowledge (EBM) and personal skills (communication, negotiation), in combination with a positive attitude towards physiologic birth, helped to realise the goal of developing protocols that supported physiological approaches to care. Our research confirms the finding that midwives feel empowered to withstand a medical approach and a non-supportive professional environment when they can rely on ‘physiological’ guidelines [33]. Because guidelines reflect the views of their creators – on care and risk – and are not just products of evidence, midwives must be involved in the development of national and local guidelines in order to insure the incorporation of their physiological orientation.

Corresponding with the findings of Porter et al. [30], characteristics of the midwife played an important role in how the protocols were used in everyday practice. Midwives with strong and positive attitudes toward the promotion of physiological birth and woman centredness invested more in empowering women to make their own choices. They applied the guidelines on a case-by-case basis instead of a “one size fits all” approach and were more willing to discuss women’s preferences with obstetricians when relevant. On the other hand, we also observed that midwives may be ‘medicalised’ by their environment [8, 35], underscoring the importance of a continuous and critical reflection on one’s attitude toward, and knowledge of, physiological birth.

The variety of factors influencing decision-making and the complex relation between them may explain the variation in intrapartum referral rates as found (inter)nationally [4–6]. Variation in clinical decisions is inevitable if care is tailored to the specific circumstances and preferences of women. However, different studies have shown that the complexity of decision-making may contribute to unwanted variation in clinical decisions, limiting a woman’s opportunity for physiological pregnancy and childbirth [5, 6, 8]. We found that the treatment of pregnant and birthing women varied between locations and between professionals within each location, as did the promotion and protection of physiological birth. Midwives are often regarded as the protectors of physiological birth and our study provides insight in how different midwives experience and execute that role in everyday clinical decision-making. Given the strong evidence that the increasing medicalisation of birth does not necessarily contribute to better outcomes for women and their babies – and may even do harm [42–47] – midwives should reconsider and strengthen their role of the protectors of physiological childbirth.

### Strengths and limitations of the study

This was a study of eleven midwives working in primary care, so our results may not be generalisable for all midwives working in primary and secondary care. However, as with all qualitative research, our goal was not statistical representation, but a rich understanding of the behaviour of our participants. It may be that social desirability influenced midwives’ responses, although in their evaluations of the interviews, all participants indicated that they felt safe to speak freely. Our study is based on self- reports. Studies using observations are required to confirm that what midwives reported is actually what they do. A strength of this study is the use of the vignettes and the Thinking Aloud Method in the context of a semi-structured interview, a combination that enabled us to obtain a broad and in-depth perspective on clinical decision-making.

## Conclusion

Although the model of EBM informs midwives’ clinical decision-making, it does not fully explain the result and process of their decisions. The professional and personal skills and the attitudes of the midwife in interaction with women and with the other members of the caregiving team and the organisation of care, play an important role in decisions that help to realise the goals of evidence-based care.

Results of this and future research on the non-clinical factors that influence the clinical decisions of midwives should be used to educate and empower (student) midwives. If midwives want to succeed in promoting and protecting physiological birth they need to understand how clinical decisions in the context of a multidisciplinary collaboration are actually made. In particular, our finding that constructive collaboration is critical for the promotion of physiological childbirth, underscores the responsibility of maternity care professionals to create an authentic collaborative culture.

## Abbreviations

BMI: Body mass index
COREQ: Consolidated criteria for reporting qualitative research
CTG: Cardiotocography
EBM: Evidence based medicine
PPH: Postpartum haemorrhage
